# Epstein-Barr Virus and Human Adenovirus Viremia in Renal Tumors Is Associated with Histological Features of Malignancy

**DOI:** 10.3390/jcm9103195

**Published:** 2020-10-02

**Authors:** Piotr Kryst, Sławomir Poletajew, Aleksandra Wyczałkowska-Tomasik, Stefan Gonczar, Maciej Wysocki, Renata Kapuścińska, Wojciech Krajewski, Wojciech Zgliczyński, Leszek Pączek

**Affiliations:** 1 Second Department of Urology, Centre of Postgraduate Medical Education, 01-809 Warsaw, Poland; piotr.kryst@cmkp.edu.pl (P.K.); stefan.gonczar@gmail.com (S.G.); 2 Department of Immunology, Transplantology and Internal Medicine, Medical University of Warsaw, 02-005 Warsaw, Poland; atomasik@wum.edu.pl (A.W.-T.); leszek.paczek@wum.edu.pl (L.P.); 3 Department of Pathology, Centre of Postgraduate Medical Education, 01-809 Warsaw, Poland; maciej.wysocki@bielanski.med.pl; 4 Department of Endocrinology, Centre of Postgraduate Medical Education, 01-809 Warsaw, Poland; renata.kapuscinska@cmkp.edu.pl (R.K.); klinendo@cmkp.edu.pl (W.Z.); 5 Department of Urology and Oncological Urology, Wrocław Medical University, 50-556 Wrocław, Poland; wojciech.krajewski@umed.wroc.pl

**Keywords:** Epstein-Barr virus, human adenovirus, polymerase chain reaction, prognosis, renal cancer

## Abstract

Background: There is growing evidence that viral infections may impact the risk and clinical course of malignancies, including solid tumors. The aim of this study was to assess the possible association of selected chronic/latent viral infections with the clinical course of renal cell carcinoma (RCC). Methods: In this prospective study we enrolled 27 patients undergoing partial or radical nephrectomy due to the histologically confirmed RCC and followed them up for one year post-operation. Isolation of the nucleic acids was performed using the NucleoSpin Tissue Kit (Macherey-Nagel, Düren, Germany) from tumor tissue and using the EZ1 Virus Mini Kit v2.0 from plasma. The number of viral copies of human adenovirus (ADV), herpes simplex virus HSV-1 and HSV-2, Epstein-Barr virus (EBV), cytomegalovirus (CMV), BK virus (BKV) and John Cunningham virus (JCV) in the tissue and plasma was assessed with real-time PCR. Results: Viral infections were diagnosed in ten patients (37.0%), including three ADV cases (11.1%) and eight EBV cases (29.6%). Infected patients tended to be significantly older (71.3 vs. 57.6 years, *p* < 0.05), more commonly presented with chronic renal disease (OR 2.4, *p* < 0.05), diabetes (OR 4.2, *p* < 0.05) and overweight (OR 2.0, *p* < 0.05). Regarding oncological data, infected patients were found to have a higher rate of high-grade cancers (OR 5.0, *p* < 0.05) and a higher rate of papillary RCCs (OR 8.3, *p* < 0.05). Status of viral infections had no influence on the clinical cancer stage, surgical procedure or survival. Conclusions: EBV and ADV infections are common in renal cancer patients and increase the risk of high-grade RCC presence. While there is no significant impact on short term survival, further studies are needed to assess the relevance of these findings in a long run.

## 1. Introduction

Renal malignancies are the sixteenth most common neoplasm worldwide [1]. The most common histological type is renal cell carcinoma (RCC), arising from renal tubular epithelium and further divided into three main subtypes, namely clear cell, papillary and chromophobe RCC [2]. Among established risk factors, one should list smoking, arterial hypertension and obesity [3,4,5]. At least 6% of RCCs present germline mutations, indicating hereditary origin [6]. Among genetic alterations related to carcinogenesis, the most common is loss of chromosome 3p and mutation of the von Hippel-Lindau gene at chromosome 3p25 [7]. Until now, viral infections and renal tissue viremia were not demonstrated as clear risk factors or modulators for RCC carcinogenesis. However, viral infections were linked to several malignancies, both in immunocompromised and immunocompetent patients.

It was assumed that as many as 15% of malignancies are caused by microorganisms [8]. Regarding urological cancers, viral infections seem to play a marginal role [9]. The strong association was proven only for human papilloma virus (HPV) and penile cancer risk [10]. However, more and more research shows a possible link between viral infections and urothelial cancer [11,12,13,14,15], prostate cancer [15,16,17] or testicular cancer [18,19]. Furthermore, the evidence behind the role of latent viral infections in carcinogenesis of RCC is growing. Some authors confirmed viral infection in RCC and suggest its role as a risk factor, a predictor of cancer histology and biological behavior or a consequence of immunocompromised tumor environment [20,21,22]. However, relation between renal cell carcinogenesis, RCC and viral infections is still to be defined.

## 2. Materials and Methods

The aim of this study was to assess the possible association of selected chronic/latent viral infections of RCC tumors with the clinical course of renal cancer.

### 2.1. Patients

In this prospective study we enrolled 27 patients undergoing the surgery due to renal tumor. Their mean age was 62.7 years, male to female ratio was 2.7:1. Twelve (44.4%) and fifteen (55.6%) patients underwent partial and radical nephrectomy, respectively. All subjects gave their informed consent for inclusion before they participated in the study. The study was conducted in accordance with the Declaration of Helsinki, and the protocol was approved by the Ethics Committee of the Medical University of Warsaw (KB/37/2017 from 7 March 2017).

Apart from the experimental methods described below, all surgical specimens were examined routinely by the urological pathologist, who eventually diagnosed RCCs in all patients, including clear cell type in 18 patients (66.7%), papillary type in 6 patients (22.2%) and chromophobe type in 3 patients (11.1%). After the surgery, patients were followed up for one year, including clinical visits and laboratory tests every three months, as well as chest-abdominal CT scans at 6 and 12 months. As one patient was lost to follow up, final survival analysis was based on 26 out of 27 patients.

### 2.2. Viruses

Before the surgery, blood samples were taken from all participants and plasma were frozen at −80 °C. After the surgery, tissue homogenates from tumor specimens were tested for the presence of human adenovirus (ADV), herpes simplex virus HSV-1 and HSV-2, Epstein-Barr virus (EBV), cytomegalovirus (CMV), BK virus (BKV) and John Cunningham virus (JCV). After diagnosing EBV and ADV infections in tissue specimens, ADV and EBV nucleic acids were sought in the corresponding plasma samples.

### 2.3. Number of ADV, HSV-1/2, EBV, CMV, BKV and JCV Virus Copies in Renal Tumor Tissue

DNA isolation from tumor tissue was performed using the NucleoSpin Tissue Kit (Macherey-Nagel, Düren, Germany), according to the manufacturer’s instructions. To a tube with about 25 mg of small pieces of tissue, 180 µL of Buffer T1 and 25 µL of Proteinase K were added. This mixture was incubated at 56 °C for 90 min. As a second step, 200 µL of Buffer B3 was added to the lysate and incubated at 70 °C for 10 min. Next, 210 µL of ethanol 99.8% was added to the lysate (POCH, Gliwice, Poland). The sample was applied to the column and centrifuged for 1 min at 11,000× *g*. To wash silica membrane, 500 µL of Buffer BW was added and centrifuged for 1 min at 11,000× *g*, then 600 µL of Buffer B5 was added to the column and centrifuged for another 1 min at 11,000× *g*. The silica membrane was dried by centrifugation for 1 min at 11,000× *g*. The column was put in a new tube and 100 µL of Buffer BE was added. It was then incubated at room temperature for 1 min and centrifuged 1 min at 11,000× *g*.

The number of ADV virus copies in the tissue was assessed with the real-time PCR method, using the primer sets and probes described previously [23]. For real-time PCR reaction we used DNA or standards, mastermix, primers and probes (sequences of primers and probes 5′–3′: GGA CGC CTC GGA GTA CCT GAG, ACA GTG GGG TTT CTG AAC TTG TT, JOE-CTG GTG CAG TTC GCC CGT GCC-TAMRA). RNase free water was used as the no template control. The reaction was run with amplification profile: 15 min at 95 °C, 45 cycles—10 s at 95 °C, 30 s at 55 °C, 15 s at 72 °C. The viral load of ADV was automatically calculated by the analyzer CFX96Dx Real Time PCR Detection System (BioRad, Hercules, CA, USA) relative to the standard curve.

The number of HSV-1/2 and EBV virus copies in the tissue was assessed with the real-time PCR method, using the diagnostic test R-gene Kit (bioMérieux, Marcy l’Etoile, France) according to the manufacturer’s instructions. For real-time PCR reaction we used DNA or standards, mastermix, primers and probes. RNase free water was used as the no template control. The reaction was run with amplification profile: 15 min at 95 °C, 45 cycles—10 s at 95 °C, 40 s at 60 °C. The viral load of HSV-1/2 and EBV was automatically calculated by the analyzer D × 96 (BioRad, Hercules, CA, USA) relative to the standard curve.

The number of CMV, BKV and JCV virus copies in the tissue were assessed with the real-time PCR method, using the diagnostic test GeneProof PCR Kit (GeneProof, Brno, Czech Republic) according to the manufacturer’s instructions. For real-time PCR reaction we used DNA or standards, mastermix, primers and probes. RNase free water was used as the no template control. The reaction was run with amplification profile: 2 min at 37 °C, 10 min at 95 °C, 45 cycles—5 s at 95 °C, 40 s at 60 °C, 20 s at 72 °C. The viral load of CMV, BKV and JCV was automatically calculated by the analyzer ABI7500 (Applied Biosystems, Foster City, CA, USA) relative to the standard curve.

### 2.4. Number of EBV and ADV Virus Copies in Plasma

Isolation of viral nucleic acids from plasma was performed using EZ1 Virus Mini Kit v2.0 (Qiagen, Hilden, Germany), according to the manufacturer’s instructions with EZ1 BioRobot device (Qiagen, Hilden, Germany). All reactions were run in automatic apparat.

Real-time PCR reaction described above for CMV, BKV and JCV diagnosis were adopted also for assessment of EBV and ADV virus copies in the plasma. As the study was focused on local viremia as a potential prognostic factor for renal cancer, only parameters that were positive in tissue were evaluated in plasma.

### 2.5. Calculation of Viral Load

The viral load of all viruses in tissue and plasma was automatically calculated by the analyzer relative to the standard curves, which were as follows:

- human adenovirus (ADV): 500, 5000, 50,000, 500,000 cp/mL,
- herpes simplex virus-1 (HSV-1): 2, 20, 200, 2000 cp/µL,
- herpes simplex virus-2 (HSV-2): 2, 20, 200, 2000 cp/µL,
- Epstein-Barr virus (EBV): 10, 100, 1000, 10,000 cp/µL,
- cytomegalovirus (CMV): 10, 100, 1000, 10,000 cp/µL,
- BK virus (BKV): 10, 100, 1000, 10,000 cp/µL,
- John Cunningham virus (JCV): 10, 100, 1000, 10,000 cp/µL.

### 2.6. Statistical Analysis

Results are presented as absolute values, percentages and mean or median values for variables with or without normal distribution, respectively. Normal distribution was tested with Shapiro-Wilk test. Levene test was applied to assess the equality of variances. For comparisons between study groups, unpaired *t*-test and Pearson test were used for quantitative and qualitative variables, respectively. A *p* value of < 0.05 was considered statistically significant.

## 3. Results

Viral sequences within tumors were diagnosed in tissue specimens from ten patients (37.0%), including eight cases of EBV (29.6%) and three cases of ADV (11.1%). In one patient concomitant EBV and ADV viral sequences were found. For all other examined infections, the results were negative. Serum tests were also negative for viral sequences in all patients. Results of viral tests are presented in Table 1.

Of note, infected patients tended to be significantly older (71.3 vs. 57.6 years, *p* < 0.05), more commonly presented chronic renal disease (70% vs. 29%, OR 2.4, *p* < 0.05), diabetes (50% vs. 12%, OR 4.2, *p* < 0.05) and overweight (80% vs. 41%, OR 2.0, *p* < 0.05). Table 2 presents a comparison of patients depending on status and etiology of viral infection.

Regarding oncological data, infected patients were found to have a higher rate of poorly differentiated cancers defined as high-grade (60% vs. 12%, OR 5.0, *p* < 0.05). Simultaneously, there was a higher rate of papillary RCC in this group of patients (50% vs. 6%, OR 8.3, *p* < 0.05). Status of viral infection had no influence on clinical stage of renal cancer or surgical procedure (partial vs. radical nephrectomy).

When analyzing separately ADV and EBV infections, only the impact on the rate of chronic kidney disease remained statistically significant for ADV (100% vs. 29%, OR 3.4, *p* < 0.05), while EBV infection increased the rate of high-grade cancers (63% vs. 12%, OR 5.3, *p* < 0.05) with simultaneous higher rate of diabetes (63% vs. 12%, OR 5.3, *p* < 0.05) and overweight (88% vs. 41%, OR 2.1, *p* < 0.05).

## 4. Discussion

With increasing evidence on the impact of a variety of viral infections on cancer development and limited data on the relationship between viral infections and RCC, we have conducted a prospective study aimed at the assessment of the incidence of selected viral infections within renal tumors. We have found that EBV and ADV tumor infections are common and are associated with different histological cancer features.

While there are some data regarding the link between EBV infection and renal malignancy, to the best of our knowledge the association of ADV with renal tumors is raised for the first time. ADV usually causes acute self-limiting infections with mild clinical symptoms within the eyes, respiratory or gastrointestinal tract. However, in some cases ADV can establish a latency within T lymphocytes [24]. For this reason, it is advised to differentiate ADV infection from disease [25].

The ADV infections and reinfections are more common and have more severe clinical course in immunocompromised patients, i.e., after organ transplantation [26,27]. We hypothesized that alterations within the immune system related to carcinogenesis can increase a risk of viral infections. This can explain a high rate of ADV presence in renal tumors, that we have noted in our study. The role of ADV infection in cancers is poorly understood and our study highlights the need of future research.

Much more is known in the field of EBV and cancer. After primary infection, EBV causes a lifelong asymptomatic latent infection within memory B lymphocytes [28]. It is assumed that 95% of the healthy adult population is infected [29]. This can be associated with latent gene heterogeneity and deletions. A special interest was focused on the loss of function of LMP1, EBNA3B, EBNA2 and B95-8 suppressor genes [30,31,32,33]. In some cases, especially in the context of immunodeficiency, such infection can promote carcinogenesis, i.e., Burkitt’s lymphoma, nasopharyngeal carcinoma, Hodgkin’s disease and others [34]. Latent EBV infection also increases the risk of gastric cancer, so called EBV-associated gastric carcinoma, which nowadays accounts for 2–20% of gastric cancer cases and is associated with a relatively good prognosis [35,36,37]. Simultaneously, EBV was detected in numerous tumors, including lymphoid, epithelial and mesenchymal tumors [34]. The first report on the causative role of EBV infection in kidney carcinogenesis in transgenic mice was published in 1997 by Törnell et al. [38].

The relation between EBV infection and renal cancer was previously reported [20,21,22,39,40]. What we did find is that EBV infection within renal tumors is frequent and associated with high-grade tumors. Shimakage et al. noted EBV infection within 100% of renal tumors [21]. On the contrary, Salehipoor et al. did not find any case of EBV infection among 49 renal cancer patients [22]. Kim et al. found that EBV virus could be a marker of sarcomatoid RCC, as it is present in tumor-infiltrating B cells due to local modulation of immune response [20]. On the other hand, Karaarslan et al. observed EBV infection in 48% of RCCs, including the presence of EBV DNA in tumor cells in 22% of cases [39]. Kang et al. noticed EBV presence in both tumor cells and tumor-infiltrating lymphocytes in 34% of RCC patients and the later phenomenon was found to be an independent prognostic factor of poor patient survival [40]. Finally, Becker et al. showed that EBV infection of renal proximal tubular cells may participate in evoking a cellular immune response that results in a damaged renal interstitium in patients with chronic interstitial nephritis [41]. Taking all these data together, it remains unclear whether EBV infection is a cause or a result of RCC development and whether the infection is specific for tumor cells, B lymphocytes, renal parenchyma or all of them.

Apart from the finding that RCCs are infected with EBV and ADV, we did show that these infections lead to a higher rate of high-grade cancers. Therefore, one can expect shorter survival in these patients as cancer grade is one the most important prognostic factors in postoperative follow-up [42,43]. This was already proven by Kang et al., who noticed significantly shorter overall survival in RCC patients and EBV infected tumor-infiltrating lymphocytes [40].

We have also noted that patients with viral infections have a higher rate of chronic renal disease. However, this fact can be at least partially attributed not to virus, but to other differences in patient characteristics, including older age, higher rate of diabetes and being overweight. All these facts are well known risk factors for renal disease. This explanation is supported by a study from Blazquez-Navarro et al., which showed that EBV has no significant impact on the risk of renal failure in patients after renal transplantation [44].

This study is not free from limitations. First, the study population is limited. However, for a pilot study with seven viruses tested, this limitation is justified to some extent. For future studies, one should plan to focus on EBV and ADV and enroll more patients. Second, as study methods clearly diagnosed or excluded viral infections in tissue homogenates, no information was gathered on whether viral genetic material comes from cancer cells, infiltrative lymphocytes or other cells. This doubt does not change substantially the clinical meaning of our findings. However, it needs to be addressed in the future. Third, the selection of tested viruses was subjective and does not rule out the importance of other viral infections in renal malignancies. Until now, the possible association between renal carcinogenesis and HPV infection [8] or hepatitis C virus infection [45] was suggested.

## 5. Conclusions

EBV and ADV viremia in RCC tumors is common and it is associated with the risk of high-grade malignancy. While there is no significant impact on short term survival, further studies are needed to assess the relevance of these findings in the long term.

## Figures and Tables

**Table 1:** Viral infections in renal cell carcinoma (RCC) tissues.

	Total Number of Positive Cases	Mean Value of the Viral Copy Numbers/uL
EBV	8	198.3 (range 29–829)
ADV	3	619.7 (range 393–867)
HSV-1	0	-
HSV-2	0	-
CMV	0	-
BKV	0	-
JCV	0	-

**Table 2:** Comparison of patients depending on status and etiology of viral infection.

Parameter	Non-Infected Patients (Controls) (*n* = 17)	Infected Patients (ADV or/and EBV Infection) (*n* = 10)	*p*-Value (Controls vs. ADV/EBV)	Patients with ADV Infection (*n* = 3)	*p*-Value (ADV Positive vs. ADV Negative Patients)	Patients with EBV Infection (*n* = 8)	*p*-Value (EBV Positive vs. EBV Negative Patients)
Percentage of women	41.2%	30.0%	>0.05	66.7%	>0.05	25.0%	>0.05
Mean age (years)	57.6	71.3	0.02	73.3	>0.05	69.9	>0.05
Mean BMI (kg/m^2^)	24.5	27.9	0.01	26.7	>0.05	28.3	0.01
Percentage of overweight patients (BMI > 25 kg/m^2^)	41.2%	80.0%	0.0499	66.7%	>0.05	87.5%	0.03
Percentage of patients with diabetes	11.8%	50.0%	0.02	33.3%	>0.05	62.5%	0.006
Percentage of patients with uncontrolled dyslipidemia	41.2%	10.0%	>0.05	0%	>0.05	12.5%	>0.05
Median white blood cell count (k/uL)	8.79	6.14	>0.05	10.04	>0.05	5.97	0.049
Median hemoglobin concentration (g/dL)	13.8	13.1	>0.05	13.3	>0.05	13.1	>0.05
Percentage of abnormal CRP values (>5 mg/L)	23.5%	30.0%	>0.05	66.7%	>0.05	12.5%	>0.05
Median creatinine serum concentration (mg/dL)	0.76	1.09	0.047	1.11	>0.05	1.08	>0.05
Percentage of patients with chronic kidney disease (GFR < 60 mL/min/1.73 m^2^)	29.4%	70.0%	0.04	100%	0.04	62.5%	>0.05
Percentage of patients with proteinuria (>30 mg/dL)	23.5%	20.0%	>0.05	33.3%	>0.05	12.5%	>0.05
Percentage of patients with hematuria (>3 erythrocytes/HPF)	41.2%	50.0%	>0.05	66.7%	>0.05	50.0%	>0.05
Percentage of patients with pyuria (>5 leukocytes/HPF)	35.3%	50.0%	>0.05	66.7%	>0.05	37.5%	>0.05
Pathological stage of cancer	pT1a–58.8% pT1b–23.5% pT2a–5.9% pT2b–5.9% pT3a–5.9% pT3b-4–0% missing–0%	pT1a–20% pT1b–20% pT2a–20% pT2b–10% pT3a–20% pT3b-4–0% missing–10%	>0.05	pT1a–0% pT1b–33.3% pT2a–33.3% pT2b–33.3% pT3a–0% pT3b-4–0% missing–0%	>0.05	pT1a–25.0% pT1b–12.5% pT2a–12.5% pT2b–12.5% pT3a–25.0% pT3b-4–0% missing–12.5%	>0.05
Histological grade of cancer	low grade–76.5% high grade–11.8% missing–11.8%	low grade–40.0% high grade–60.0% missing–0%	0.01	low grade–33.3% high grade–66.7%	>0.05	low grade–37.5% high grade–62.5%	0.02
Histological RCC subtype	clear cell–76.5% papillary–5.9% chromophobe–17.6% other–0%	clear cell–40.0% papillary–50.0% chromophobe–0% other–10.0%	0.004	clear cell–33.3% papillary–66.7% chromophobe–0% other–0%	>0.05	clear cell–50.0% papillary–37.5% chromophobe–0% other–12.5%	>0.05
Surgical procedure	partial nephrectomy–47.1% radical nephrectomy–52.9%	partial nephrectomy–40.0% radical nephrectomy–60.0%	>0.05	partial nephrectomy–33.3% radical nephrectomy–66.7%	>0.05	partial nephrectomy–37.5% radical nephrectomy–62.5%	>0.05
12-month cancer recurrence rate	12.5%	10.0%	>0.05	0%	>0.05	12.5%	>0.05
